# Land-based versus aquatic resistance therapeutic exercises for older women with sarcopenic obesity: study protocol for a randomised controlled trial

**DOI:** 10.1186/1745-6215-14-296

**Published:** 2013-09-16

**Authors:** Karina Simone de Souza Vasconcelos, João Marcos Domingues Dias, Marília Caixeta de Araújo, Ana Cisalpino Pinheiro, Marcela Machado Maia, Rosângela Corrêa Dias

**Affiliations:** 1 Escola de Educação Física, Fisioterapia e Terapia Ocupacional, Departamento de Fisioterapia, Universidade Federal de Minas Gerais, Avenida Presidente Antônio Carlos, 6627 Campus Pampulha, Belo Horizonte, MG, CEP 31270-901 Brazil

**Keywords:** Older people, Women, Sarcopenic obesity, Resistance exercises

## Abstract

**Background:**

Sarcopenic obesity is a health condition that combines excess adipose tissue and loss of muscle mass and strength. Sarcopenic obesity predisposes to more functional disabilities than obesity or sarcopenia alone. Progressive resistance exercises are recommended for older people as a potential treatment for sarcopenia and also for obesity. However, there is a lack of evidence indicating which programmes are best applied to older people, and no studies have investigated their effects on sarcopenic obese people. The aims of this protocol study are to investigate and compare the efficacy of land-based and aquatic resistance exercise programmes on improving muscle performance, functional capacity and quality of life of older women with sarcopenic obesity.

**Methods/Design:**

This is a protocol study for a parallel randomised controlled clinical trial. Eligible participants are older women (≥65 years) with a body mass index ≥30 kg/m ^2^ and hand grip strength ≤21 kg force. A total sample of 36 participants will be randomly allocated to one of the intervention groups in blocks of three: land-based, aquatic or control. Each intervention group will undergo 2-week sessions of a 10-week therapeutic exercise programme for strength, power and endurance training of the lower-limb muscles. Participants in the control group will not participate in any strengthening activity for lower limbs and will receive telephone calls once a week. Baseline and final evaluation of outcomes will encompass muscle performance of the lower limbs assessed by an isokinetic dynamometer; functional tests of usual walking speed, maximal walking speed (shuttle walking test), stair speed and the Short Physical Performance Battery; and health-related quality of life (Medical Outcomes Study Short Form Questionnaire – SF-36). Data collectors will be blinded to randomisation and will not be in touch with participants during the interventions.

**Discussion:**

This study is the first randomised controlled trial designed to evaluate resistance exercises in older patients with sarcopenic obesity. If our hypothesis proves correct, both intervention programmes will be effective, with the land-based exercises conferring better results in muscle performance.

**Trial registration:**

Registro Brasileiro de Ensaios Clínicos: RBR-9p5q67

## Background

Ageing is generally followed by physiological changes in body composition. Essentially, ageing involves a gradual loss of muscle mass, a process called sarcopenia, and an increased amount of adipose tissue, which may lead to obesity [1,2]. The process of sarcopenia also encompasses a worsening of muscle function, characterised by weakness or low physical performance [3]. Obesity prevalence among older people has reached levels comparable with young adults, especially among women [4]. In extreme cases, excess adipose tissue and muscle mass loss may be combined in a condition called sarcopenic obesity [5,6]. According to diagnostic criteria, the prevalence of sarcopenic obesity may reach 12% among individuals aged over 60 years [7,8]. Disability is more common among older people with sarcopenic obesity than those with either condition separately [9-11].

Progressive resistance exercises have been widely recommended to older people as a potential treatment for sarcopenia because they improve muscle performance and functional capacity [12-14]. There is a lack of evidence indicating which programmes are optimal for older people because resistance exercises are often applied with the same parameters as followed for young people [15,16]. In older people with obesity, these exercises can produce weight loss and muscle mass gains, improving body composition and preventing the worsening of sarcopenia [5,13,17]. In addition, resistance exercises may stem the pathological progression of sarcopenic obesity by reducing the levels of inflammatory cytokines [18-20].

Using an aquatic environment may be a suitable way for older people to exercise, especially for safety and comfort. For obese people, water-based exercises can minimise joint impacts and facilitate movements. Although aquatic-based exercises are common in clinical practice, there is little evidence of the effects of these exercises, especially because of methodological problems and small sample sizes in previous studies [21]. Some studies found significant benefits of aquatic exercises in muscle performance and functional capacity of older people, but they combined aerobic and resistance training programmes [22,23]. Those authors suggest that the strength gains associated with aquatic exercises are smaller than those resulting from land-based programmes.

Our research group has found evidence that resistance exercise programmes can improve muscle performance and reduce the inflammatory state of older people [24,25], but we were unable to find any studies that investigated the effects of progressive resistance exercises in sarcopenic obese people. Additionally, there is a lack of evidence about the effects of aquatic-based resistance training in general [15,21].

This study therefore seeks to answer the following questions: Do land-based and aquatic resistance exercise programmes improve muscle performance, functional capacity and quality of life of older women with sarcopenic obesity? Which of the two exercise programmes is more effective?

## Methods/Design

### Study design

This study is a randomised clinical trial with two intervention groups and a control group. This study was approved by the Ethics Research Committee of Federal University of Minas Gerais (Universidade Federal de Minas Gerais), Belo Horizonte, Brazil, under number ETIC 0172.0.203.000-11.

### Sample size

**Figure 1:**
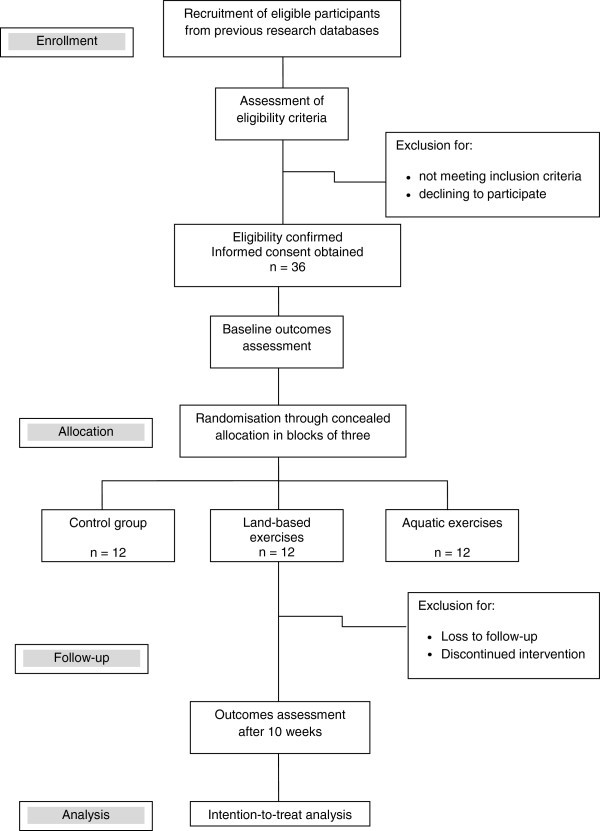
Flow diagram of the study protocol.

The effect size of the interventions was estimated at *f* = 0.20, based on previous studies of similar exercise programmes and the target population [15,26,27] and a pilot study conducted with 12 volunteers. The sample size was calculated assuming a mixed design of repeated-measures analysis of variance in the programme G* Power, version 3.1.2 [28], considering α = 0.05, β = 0.80, three groups and a correlation of 0.8 between the two repeated measures, with a resultant total of 30. Adding a possibility of 15% missing participants, the final sample size was calculated as 36, with 12 subjects per group. Figure 1 shows the flow diagram for participants in this study.

### Study population

Volunteers for this study will be recruited from the database of our research group on aging. The general inclusion criteria for all of the older people enrolled in this database are community-dwelling people aged 65 years or older. The exclusion criterion is to be bedridden. The professor in charge of the research group will make available the information about sociodemographic and anthropometric characteristics of the registered older people. We will then search for obese older women. The potential volunteers will be contacted by telephone and invited to participate in this research project.

Eligible participants are older women (≥65 years) with a body mass index ≥30 kg/m^2^[29] and hand grip strength ≤21 kg force [30].

Exclusion criteria are: physical, sensory or cognitive disabilities that prevent testing or exercising; inability to adapt to an aquatic environment; cardiovascular, articular or metabolic disease in the acute phase or with unbalanced clinical symptoms; urinary or faecal incontinence; contagious skin diseases, ulcers or open wounds; currently attending physical therapy treatment for lower limbs; and underwent lower-limb surgery or had a lower-limb fracture in the 12 months before enrolment.

### Primary outcomes

Muscle and functional performance are the primary outcomes of this study. Muscle performance of lower limbs will be assessed using an isokinetic dynamometer (Biodex System 3 Pro; Biodex Medical Systems Inc., Shirley, NY, USA) by measuring the strength and power of the hips and knees. The functional capacity of locomotion will be assessed through tests of usual walking speed, maximal walking speed, stair speed and the Short Physical Performance Battery.

### Secondary outcomes

Health-related quality of life will be assessed by the Brazilian version of the Medical Outcomes Study Short Form Questionnaire – SF-36 [31].

### Procedures

First, eligible older women will be informed of the study’s objectives and procedures of datacollection methods. The benefits and risks involved in voluntary participation will be stated. A researcher will provide this information in an interview, using an information sheet. If the volunteer decides to participate in the study, a written consent statement will be obtained.

The Mini-Mental State Examination will then be applied to assure cognitive capacity for participation in the study. Volunteers with a score <17 will not be included in the study [32].

A clinical questionnaire was prepared to collect sociodemographic, anthropometric and clinical data to characterise the study sample. Participants will be classified as frail, prefrail or nonfrail according to the criteria proposed by Fried and colleagues [30]. The Brazilian version of the Human Activity Profile will be applied to characterise participants into three categories: inactive, moderately active and active [33]. The Geriatric Depression Scale short form – GDS-10 [34] will be used to assess possible depressive symptoms in the volunteers.

After an initial interview, each participant will undergo the functional tests in a random order. In each test, the presence of pain in the lower limbs and the rate of perceived exertion [35] will be registered. The Brazilian version of the Short Physical Performance Battery will be used for tests of balance, walking speed and rising from a chair [36]. The best time of two trials in each of these tasks will recorded, and participant performance will be classified on a 4-point scale for each test [37].

The shuttle walking test will be conducted to calculate maximum walking speed [38]. In this test, the volunteer must walk a 10-metre course at progressively higher speeds, trying to make this distance before a beep sounds. During the test, the beeps will become closer every minute, leading the volunteer to walk at an ever-increasing rate. The initial velocity is 0.5 m/second, which increases by 0.17 m/second every minute, for a maximum duration of 12 minutes. The test is stopped when the individual cannot reach the end of the course before the beep in two successive trials.

Locomotion on stairs will be tested on a flight of 10 stairs with a handrail. In the first test, volunteers will be instructed to climb the stairs in their usual way, going up and down at once. The second test is to climb the flight of stairs as quickly as possible, stopping at the top of the stairs. Each test is performed twice, and the best time is recorded to calculate the usual stair speed and maximum stair speed as the ratio between the height of the stairs and the time spent to accomplish the task [39].

To prevent potential fatigue, muscle performance tests will be conducted on a day after the interview and functional tests. For tests of knee flexors and extensors, the manufacturer’s protocols (Biodex System 3 Pro; Biodex Medical Systems Inc.) will be followed in the sitting position. For hip muscles (flexors/extensors and the abductor/adductor groups), the tests will be performed in a standing upright position, using a stabilising device specially built for this purpose [40]. The testing order of muscles will be randomised by drawing lots. For each muscle, two different tests will be conducted. First, a test of five maximum concentric repetitions at an angular speed of 60°/second will be conducted to calculate strength as the total work (Joules) and peak torque (Newton-meters). Next, a test of 15 repetitions at a speed of 180°/second for knee muscles and 120°/second for hip muscles will be performed to calculate power as the work done per unit time, expressed in Watts [41]. All measures will be normalised to body weight.

Data collectors will be blinded to the participants’ later randomisation into groups.

### Randomisation

After complete baseline assessment, a randomisation sequence will be created using a computer-generated list of random numbers in block sizes of three. The allocation sequence will be concealed from the researchers enrolling and assessing participants, since it will be performed by an external researcher. The allocation of each participant will then be communicated to the researchers involved in the exercise programmes and to those responsible for monitoring the control group.

### Interventions

Each intervention group will undergo a therapeutic exercise programme designed to improve the strength, power and endurance of lower-limb muscles. Independent researchers who did not participate in the data collection will conduct the exercise sessions.

The exercise sessions will be done in groups of six volunteers, accompanied by at least two researchers. Each programme will last 10 weeks, with 1-hour sessions twice a week. Frequency of attendance and adverse clinical events that occur during the sessions will be registered on a daily record sheet.

The exercise programmes were designed to follow recommended principles of prescription and progression of resistance training for older adults in general and in the aquatic environment [13,42]. Researchers conducting the training sessions will guide the exercise execution with abdominal contraction, spine stabilisation and correction of lower-limb alignment and upright stance. The guidance will include verbal, tactile and visual information to ensure the correct muscle contraction demanded by the exercises.

Each session will comprise a 5-minute walk for warm-up and lower-limb exercises for stretching and strengthening, with a resting time of 30 seconds between sets and 1 minute between exercises. Stretching exercises will comprise posterior, anterior, lateral and medial lower-limb muscles, performed for 60 seconds in each leg. Strengthening exercises will be performed in open and closed chains of lower limbs.

In the first 4 weeks of the intervention, both land-based and aquatic programmes will emphasise resistance exercises for strengthening muscles, with concentric and eccentric movements performed at a low speed. Beginning in the fifth week of training, participants will be instructed to add increasing speed to each exercise, focusing on muscle power.

### Land-based exercise programme

Prior to the first land-based exercise session, participants will be tested in a one-repetition maximum test (1-MR) of knee extensors and flexors [41]. This 1-MR will be performed again after 4 weeks of the intervention.

The training programme of land-based resistance exercises will be conducted as follows.

In weeks 1 and 2, exercises will be performed in the supine (hip flexion), lateral (adduction and abduction), prone (hip extension and knee flexion), sitting (knee extension) and upright positions (mini-squat): knee exercises with two sets of 12 repetitions at 50% of 1-MR; hip exercises with two sets of 10 repetitions at 1 kg load; and mini-squats in two sets of 10 repetitions. Both concentric and eccentric movements will be performed at a slow speed.

In weeks 3 and 4, the same positioning will be used for exercises, increasing knee loads to 75% of 1-MR and hip loads to 2 kg. The same number of sets and repetitions will be performed, at low speeds.

In weeks 5 and 6, exercises will be performed in the supine (adduction), prone (abduction and knee flexion), upright (hip flexion and extension) and sitting positions (knee extension). After a new 1-MR testing, loading will be changed to 40% of 1-MR for knees and remaining at 2 kg for hips. Participants will maintain the same number of sets and repetitions, performing concentric movements at a high speed. For mini-squats, one set will be performed at high speed.

In weeks 7 and 8, the same positioning and number of sets and repetitions will be maintained, but increasing the loads to 60% of 1-MR for knees and 3 kg for hips. Concentric and eccentric movements will be performed at higher speeds.

In weeks 9 and 10, positioning and loading will be maintained, with the addition of one set for each exercise, performed at high speeds.

### Aquatic exercise programme

Aquatic training will be conducted in a hydrotherapy pool of 12 m^2^ with access ramps and handrails. Water temperature will be maintained at 32°C. Prior to the first exercise session, volunteers will participate in one training session for familiarisation with the aquatic environment.

Exercises will be performed in an upright position, with water up to the xiphoid process of the volunteers. Open-chain exercises will include hip and knee flexion and extension, adduction and abduction. Closed-chain exercises will include a movement of combined hip and knee extension over a flotation accessory. Overloading in the aquatic programme will be achieved by increasing the velocity and duration of movements and adding flotation accessories.

The training programme of aquatic resistance exercises will be conducted as follows.

In weeks 1 and 2, two sets of 30 seconds of each exercise will be performed without flotation accessories for open-chain movements. Only concentric movements will be performed at a high speed.

In weeks 3 and 4,the same sets and movements will be performed with flotation accessories on lower-limb extremities for increased loading.

In weeks 5 and 6, two sets of 60 seconds of each exercise will be performed, with concentric and eccentric movements at high speeds, without accessories for open-chair exercises.

In weeks 7 and 8, two sets of 60 seconds of exercises will be performed with flotation accessories on lower-limb extremities. Concentric and eccentric movements will be performed at higher speeds.

In weeks 9 and 10, three sets of 60 seconds of exercises will be performed with flotation accessories and both concentric and eccentric movements at higher speeds.

### Control group

Volunteers randomised to the control group will be instructed not to participate in physical activities that include strengthening exercises for the legs. During the 10 weeks of interventions, they will receive telephone calls once a week. In these telephone calls, a trained researcher will interrogate them about events of health problems, temporary disabilities or pain in the lower limbs, recording any clinical complications or medication use. The researcher will reinforce their obligation to maintain normal daily activities and avoid strengthening exercises. After this period, these volunteers will have the choice to participate in one of the two types of interventions in the same programme as the other groups.

### Statistical analysis

For data analysis, we will use the Statistical Package for the Social Sciences version 15.0 (SPSS Inc., Chicago, DE, USA). The baseline characteristics of the sample will be described in frequencies or measures of central tendency and dispersion. The effects of exercise programmes will be evaluated by intention-to treat analysis. The number needed to treat for the study outcomes will be calculated considering values of minimum clinically important difference as positive effects. Repeated-measures analysis of variance for mixed design and two factors will be conducted with preplanned contrasts for each group of outcomes. If necessary, correspondent nonparametric tests will be used. The results will be described as 95% confidence intervals and considered significant at *P*<0.05.

## Discussion

To the best of our knowledge, this is the first randomised controlled trial designed to evaluate the benefit of resistance exercises for older people with sarcopenic obesity. Considering the obesity epidemic and sedentary habits of western populations in general, it is essential to find interventions that can address both problems. We chose to select only older women because of their greater longevity and higher risk of functional disabilities.

Providing assurance that rehabilitation techniques are really effective in an evidence-based way is important. To achieve this, we developed a study design using an appropriate power calculation to estimate the sample size. This clinical trial will provide substantial information about the effects of a common practice in the rehabilitation field. Exercise interventions are considered a nonpharmacologic and noninvasive technique that can improve functional capacity and prevent or reverse disabilities. If our hypothesis is correct, both intervention programmes will be effective, and the land-based intervention will produce better results for muscle performance and functional capacity. We will also investigate the effects on health-related quality of life.

Following the CONSORT Statement recommendations for randomised trials of nonpharmacologic treatments, we described our intervention protocols in detail, including all qualitative and quantitative components. Training and constant supervision will guarantee the programmes’ standardisation. In the interventions proposed in this protocol, it is not possible to blind participants or those administering the interventions. Researchers involved in outcomes assessment will thus be blinded to participant allocation and will have no contact with participants besides evaluation tests. Any harm or adverse events will be recorded and reported. Important to note is that it is completely feasible to carry out the exercise programmes proposed in this study in clinical settings. Our results will be analysed and published after discharge of the last randomised patient.

## Trial status

This project started in July 2011 with the pilot study. Volunteers started to enter the trial in January 2012. Participant recruitment will most probably be completed after 2 years.

## Abbreviations

1-MR: One-repetition maximum test.

## Competing interests

The authors declare that they have no competing interests.

## Authors’ contributions

KSSV, JMDD and RCD were responsible for the study conception and implementation. JMDD is the scientific lead of the study and has raised funds for this work. RCD contributed to development of the research protocol. KSSV is responsible for managing the project. MCA, ACP and MMM helped in the development of the research protocol and drafting of this paper. KSSV and JMDD performed the writing and final corrections of this paper, with contributions from RCD. All authors approved the final manuscript.
